# Mechanical Behavior of Entangled Metallic Wire Materials under Quasi-Static and Impact Loading

**DOI:** 10.3390/ma12203392

**Published:** 2019-10-17

**Authors:** Yiwan Wu, Lei Jiang, Hongbai Bai, Chunhong Lu, Shangzhou Li

**Affiliations:** 1 Engineering Research Center for Metal Rubber, School of Mechanical Engineering and Automation, Fuzhou University, Fuzhou 350116, China; jiangleixw@163.com (L.J.); bhbk11@sina.com (H.B.); lishangzhoufzu@163.com (S.L.); 2 Department of Automotive Engineering, Hebei College of Industry and Technology, Shijiazhuang 050091, China; lchzxy2006@sina.com

**Keywords:** entangled metallic wire material, porous material, low velocity impact, quasi-static and impact behavior, stiffness and damping properties

## Abstract

In this paper, the stiffness and damping property of entangled metallic wire materials (EMWM) under quasi-static and low-velocity impact loading were investigated. The results reveal that the maximum deformation of the EMWM mainly depends on the maximum load it bears, and that air damping is the main way to dissipate impact energy. The EMWM can absorb more energy (energy absorption rate is over 60%) under impact conditions. The EMWM has excellent characteristics of repetitive energy absorption.

## 1. Introduction

Entangled metallic wire material (EMWM) is a kind of porous damping material made from various metallic wires by coiling, weaving, and molding [1]. Although some researchers prefer to use the term metal rubber (MR) [2,3,4,5] or metal wire mesh [6,7,8], the manufacturing processes of these materials are highly similar, so they are the same kind of material [9]. Because of its excellent specific stiffness, energy absorption and environmental adaptability, EMWM has triggered numerous research projects in recent years. Cao et al. [10] designed an EMWM isolator between a satellite and carrier rocket system to absorb vibrations. Ma et al. [11] investigated the compressive and dissipative behavior of metal rubber under constraints. Ma et al. [12] also studied the mechanics of shape memory alloy metal rubber. Xu et al. [13] focused on the quasi-static compressive mechanical properties of metal rubber and established a constitutive relation model of metal rubber under a unidirectional compressive load. Yang et al. [14] investigated the vibration reliability characterization and damping capability of metal rubber in the non-molding direction.

In extreme environments (such as high-low temperatures, strong impact, salt spray corrosion and nuclear radiation), traditional polymer materials cannot meet the demand for vibration reduction and shock mitigation. Porous metal materials have good energy absorption characteristics, a stable shape, and high-low temperature resistance. They can be used to replace traditional polymer materials in these extremely harsh environments. Honeycomb aluminum and aluminum foam are two kinds of commonly used porous materials for energy absorption and vibration reduction, and have been investigated extensively. Li et al. [15] investigated the dynamic response of aluminum honeycomb sandwich panels under foam projectile impact, and presented that the deformation/failure modes of sandwich panels were sensitive to the impact velocity and density of aluminum foam. Zhang et al. [16] presented a numerical method for the ballistic performance prediction of the sandwiched open cell aluminum foam under hypervelocity impact.

Common porous metal materials, including honeycomb aluminum and metal foam, are “hard frame porous metal materials”. The internal cell elements of these “hard frame” materials are rigidly connected (thin rod, thin wall). When these “hard frame” materials are subjected to heavy loading, plastic deformation will occur to absorb vibration/impact energy. These “hard frame” materials cannot absorb energy repeatedly due to irreversible plastic deformation. However, the EMWM is a kind of “open-cell soft frame porous metal material”. Its internal spiral wires hook into each other to form a porous cavity, which is filled with air. The internal cell elements of this “soft frame” material are sliding connected. When this “soft frame” material is subjected to heavy loading, a part of the energy will be stored in the form of elastic potential energy, the other part will be dissipated in the form of sliding friction, and the pores inside the EMWM will become smaller. When the external force is released, the stored elastic potential energy will return the EMWM to its original state, at the same time, the wire helixes will slide against each other to further dissipate the vibration/impact energy, and the pores inside the EMWM will return to their original locations.

Unlike quasi-static, uniaxial load-deflection tests, a few papers have focused on the impact behavior of EMWM. Liu et al. [17] investigated the impact behavior of sintered entangled steel wire materials with different porosity levels. Note that the entangled steel wire material was sintered in a vacuum furnace at 1623 K for 30 min. In essence, this material is also a hard frame material. Liu et al. stated that the main contribution to the total absorbing energy of the sintered specimens arises from the energy for plastic bending, bulking, rotating, yielding, densification and the fracturing of pore edges, as well as the breaking (or avulsion) of sintering points. Guérard et al. [1] investigated the influence of strain rate on the mechanical behavior of a single wire entangled material using a standard Hopkinson bar device, and confirmed the good suitability of EMWM for shock absorption. However, the mechanism of shock absorption was not analyzed.

EMWM can be regarded as a kind of two-phase porous damping material consisting of metal wires and air. In previous research, due to a low loading rate or small deformation, the effect of air on the mechanical properties of EMWM has not attracted the attention of researchers. This paper will investigate the mechanical behavior of EMWM under low-velocity impact, and interpret the mechanism of shock absorption with consideration of the friction between wire helixes and air damping.

## 2. Materials and Methods

### 2.1. Fabrication of the Entangled Metallic Wire Material

**Figure 1:**
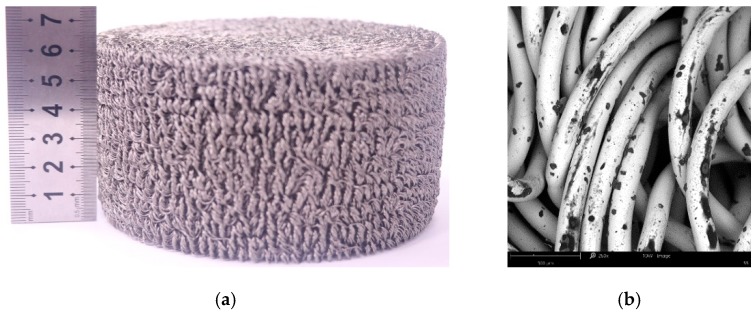
Picture and scanning electron microscope (SEM) image of EMWM. (**a**) Picture; (**b**) SEM image (260×).

The EMWM specimens were manufactured using austenitic stainless steel wires 304 (0Cr18Ni9) with a diameter of 0.3 mm via a three-step process [1,18]. First, the ordinary metal wire was encircled into a tight wire helix; second, the wire helix was stretched and weaved in a crisscross pattern to obtain a rough porous base material of EMWM; third, the rough porous base material was placed into a pre-designed mold and then was formed in a device by applying a compressive force. Figure 1 presents the picture and scanning electron microscope (SEM) image of EMWM. As shown in Table 1, to assess the repeatability of the results, 5 specimens were prepared with the same preparation parameters.

### 2.2. Experimental Devices and Methods

To compare the mechanical behavior of EMWM under quasi-static and impact loading, a series of quasi-static tests were carried out using a WDW-T200 electronic universal testing machine, and a series of low-velocity impact tests were conducted using a drop hammer. The WDW-T200 was manufactured by Jinan Tianchen Testing Machine Manufacturing Co., Ltd. (Jinan, China). Its maximum compression force is 200 kN. The drop hammer was a self-designed device. In this drop hammer system, a piezoelectric dynamic force sensor (YX-100T, Yangzhou, China) and a magnetic sensor (MTS-H25C, GIVI, Nova Milanese, Italy) were used to measure impact force and deformation, respectively. The mass of the hammer was 76 kg.

The WDW-T200 was used in displacement control mode with different speeds (0.1 mm/min, 10 mm/min, 50 mm/min and 100 mm/min). The maximum force of each quasi-static test was 200 kN. The impact velocity was set as 2 m/s, 3 m/s, 4 m/s, 5 m/s, 6 m/s and 7 m/s, respectively.

For the quasi-static test, loss factor (*η_s_*) [19] and average stiffness ($\bar{k}$) were used to evaluate the damping and load-bearing capacity of EMWM under static loading, and are commonly defined as Equations (1) and (2).

(1) $$\eta_{s}=\frac{\Delta W}{\pi \left(W-\frac{\Delta W}{2}\right)}$$

where *W* is the maximum deformation energy stored, Δ*W* is the dissipated energy.

(2) $$\bar{k}\;=\;\frac{F_{\max}}{X_{\max}}$$

where *F*_max_ is the maximum force, *X*_max_ is the maximum deformation.

For the impact test, energy absorption rate (*η_i_*) and average stiffness ($\bar{k}$) were used to evaluate the damping and load-bearing performance of EMWM under impact loading.

(3) $$\eta_{i}=\frac{v_{i}^{2}-v_{o}^{2}}{v_{i}^{2}}$$

where *v_i_* is the speed at which the hammer is in contact with the EMWM, *v_o_* is speed at which the hammer and the sample are separated.

## 3. Results and Discussion

### 3.1. Effect of Quasi-Static Velocities

**Figure 2:**
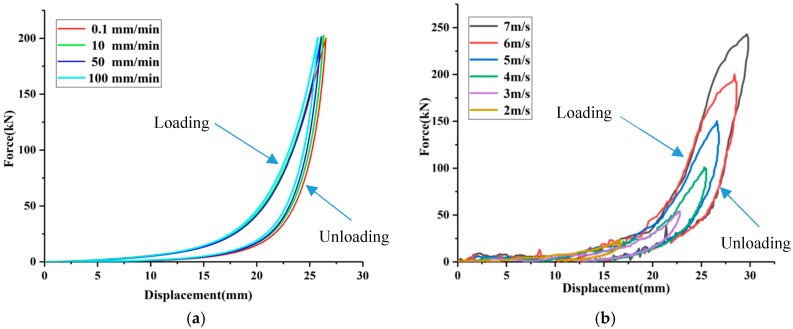
Force-displacement curves of EMWM under different quasi-static loading velocities. (**a**) Quasi-static test results; (**b**) Low-velocity impact results.

Figure 2a presents the force–displacement curves of EWMW under different quasi-static velocities (0.1 mm/min, 10 mm/min, 50 mm/min and 100 mm/min). Due to sliding friction between the adjacent wire helixes, the force during the loading/unloading process is different. Table 2 summarizes the loss factor and average stiffness for the results shown in Figure 2a.

It is noted that with the increase of quasi-static loading velocity, the loss factor decreases significantly (24.8%). The lower the loading velocity, the more sufficient the slip between the wire helixes, thus the more energy dissipated by dry friction. This result is in agreement with the results obtained in the quasi-static and low-frequency dynamic tests by Zhang et al. [7], Ertas et al. [20] and Chandrasekhar et al. [21]. However, with the increase of loading velocity, the increase of average stiffness is less than 3%. It is obvious that average stiffness is only related to the magnitude of deformation, but not to the speed of quasi-static deformation. During these quasi-static tests, the loading and unloading speeds were relatively slow, and the air inside the EMWM can be extruded and inhaled slowly. Therefore, the air damping is not obvious under quasi-static loading.

### 3.2. Effect of Impact Velocity

Figure 2b shows the force-displacement curves of EWMW under different impact velocities. It is observed that the force-displacement curves of the EMWM subjected to impact loading are similar to that subjected to quasi-static loading. Obviously, the higher the impact velocity, the larger the maximum impact loading. It is important to note that the energy dissipated by the EMWM is increased with impact velocity. Moreover, the unloading curves return to the origin. This indicates that the EMWM can absorb impact energy repeatedly.

**Figure 3:**
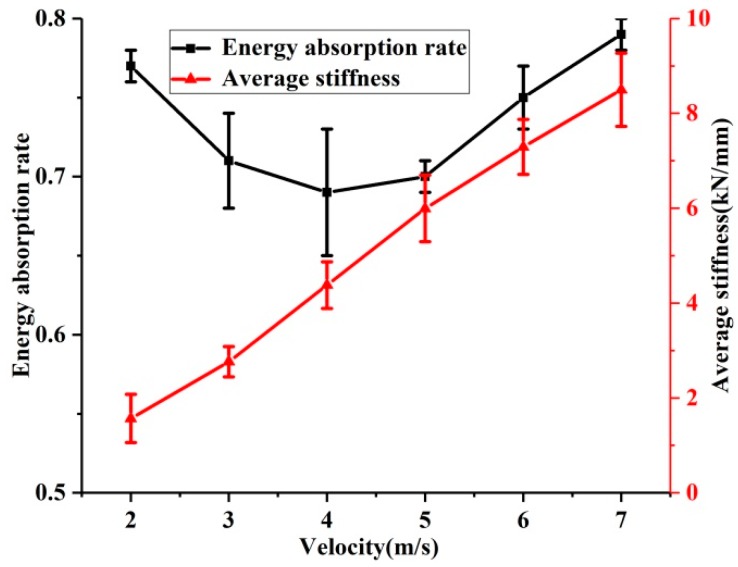
Energy absorption rate and average stiffness of the EMWM under different impact velocities.

Figure 3 presents the mean value and standard deviation of the energy absorption rate and average stiffness of the EMWM under different impact velocities. There is a significant increase in average stiffness with increase in impact velocity. There are three types of interaction (non-contact, slip, and stick) between the wire helixes in the EMWM during compression [22]. The effective stiffness for slip and stick is greater than that for non-contact. As compression load increases, the porosity of the EMWM becomes smaller, and the interaction type between the adjacent wire helixes changes (from non-contact to slip, from slip contact to stick contact). This indicates that the non-contact status occupies a smaller percentage and the number of contact points increases. The stiffness characteristic of EMWM is gradually similar to solid structures.

It can be seen in Figure 3 that the energy absorption rate decreases first and then increases at the critical point of 4 m/s. There are two reasons for this phenomenon. First, as the impact velocity increases, due to insufficient friction, the efficiency of energy dissipation through friction will be reduced. Second, as the deformation speed increases, the air damping inside the EMWM will be greater. As illustrated above, the EMWM can be regarded as a kind of two-phase porous damping material. Air is viscous and its resistance is proportional to the square of the relative velocity of the air and metal wires. As the impact velocity increases, the air damping will increase sharply. Therefore, when the impact velocity is small, friction damping plays a major role in energy absorption; when the impact velocity is large, air damping plays a major role in energy absorption.

**Figure 4:**
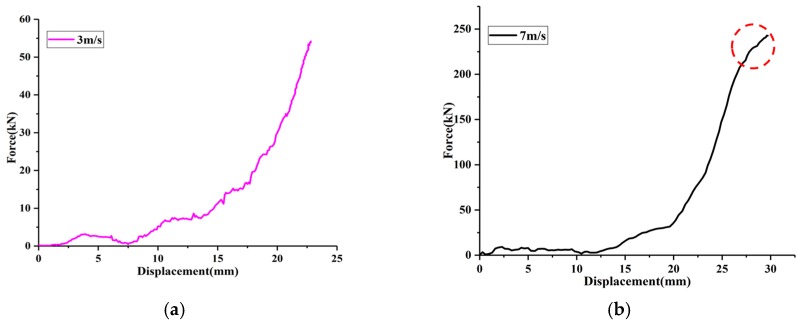

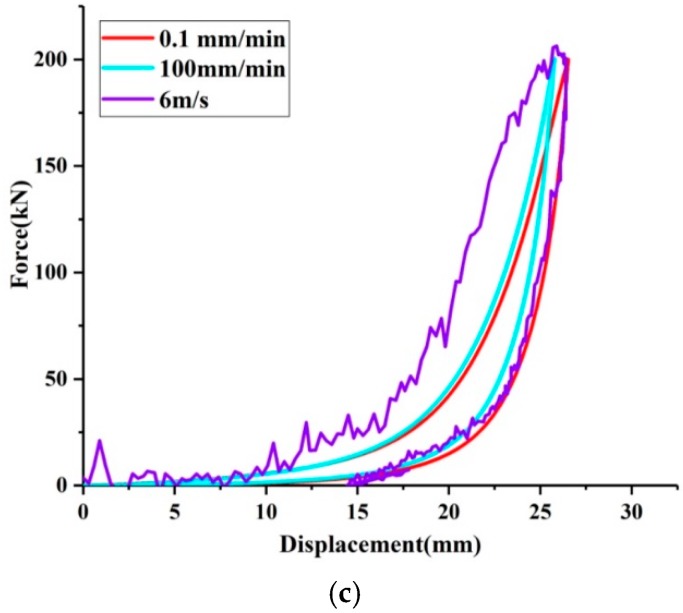
Comparison of force-displacement curves of EMWM under different loading velocities. (**a**) Impact velocity is 3 m/s; (**b**) Impact velocity is 7 m/s; (**c**) Force-displacement curves of EMWM under a loading velocity of 0.1 mm/min, 100 mm/min and 6 m/s.

To analyze the influence of air damping on stiffness, two force-displacement curves in the loading process are shown in Figure 4. It is noted that the shape of the curve with low impact velocity (3 m/s) is similar to that of the quasi-static test. The slope of the force-displacement curve under low-velocity impact (3 m/s) increases with the increase of deformation (Figure 4a). The reason for this is that the EMWM has weak air damping at a lower impact velocity. However, before reaching the maximum deformation (red dotted circle in Figure 4b), the slope of the force-displacement curve with a relatively high impact velocity (7 m/s) will decrease with the increase of deformation. In the case of a relative-high velocity impact loading, air damping is very significant. During impact loading, because impact energy is absorbed, the deformation velocity of the EMWM will become slower until it reaches maximum deformation. This means that air damping will gradually decrease until it is zero.

A comparison of an impact hysteresis loop and a quasi-static one is shown in Figure 4c. Considering that the maximum test force in the quasi-static test was 200 kN, the test result of the initial impact velocity of 6 m/s with a maximum test impact force of about 200 kN in the impact test was selected and compared. It can be seen that the impact hysteresis loop is thicker. This indicates that the EMWM will dissipate more energy in the form of friction damping and air damping under impact conditions. It is noted that the maximum force and maximum displacement of the EMWM under quasi-static and impact loading are almost the same.

The results of the quasi-static test in this paper are consistent with those of previous quasi-static tests, and the same conclusions are obtained. Friction energy dissipation will decrease with the increase of the loading rate due to insufficient sliding friction of the adjacent wire helixes. This means that the faster the loading speed, the less energy dissipated by sliding friction. However, with the increase of impact velocity, the dissipation of shock energy increases. The effect of air damping on the energy dissipation of EMWM under impact loading should not be ignored. The conduct dropping hammer test under different vacuum conditions can evaluate the effect of air damping on the energy dissipation of EMWM, and can better interpret the mechanism of shock absorption. While the existing test system does not yet have this function, vacuum testing for quantitative analysis can be carried out in the future to improve the impact energy absorption mechanism of EMWM.

## 4. Conclusions

This paper investigates the mechanical behavior of EMWM under quasi-static and impact loading, and analyzes the mechanism of shock energy absorption. Three conclusions can be drawn as follows:

(1) The maximum deformation of the EMWM mainly depends on the maximum load it bears.
(2) With the increase of impact velocity, the effect of air damping inside EMWM on its mechanical properties will be more significant.
(3) The EMWM has excellent characteristics of repetitive shock energy absorption.

## Figures and Tables

**Table 1:** Dimensions and structure parameters of EMWM specimens.

Specimen Number	Mass (g)	Diameter (mm)	Height (mm)	Relative Density	Wire Diameter (mm)
EMWM _1	1355	120	61.5	0.242	0.3
EMWM _2	1356	120	60	0.252	0.3
EMWM _3	1354	120	62	0.243	0.3
EMWM _4	1357	120	61	0.248	0.3
EMWM _5	1356	120	58	0.261	0.3

**Table 2:** Loss factor and average stiffness under different quasi-static loading velocities.

Velocity (mm/min)	Loss Factor	Average Stiffness (kN/mm)
0.1	0.1929	7.5409
10	0.1794	7.6622
50	0.1657	7.6800
100	0.1493	7.7209
